# Understanding reliability of the observer-reported communication ability measure within Angelman syndrome through the lens of generalizability theory

**DOI:** 10.1186/s41687-024-00725-9

**Published:** 2024-05-14

**Authors:** Dandan Chen, Christina K. Zigler, Li Lin, Nicole Lucas, Molly McFatrich, Jennifer Panagoulias, Allyson Berent, Bryce B. Reeve

**Affiliations:** 1 Center for Health Measurement, Department of Population Health Sciences, Duke University School of Medicine, Durham, NC USA; 2 The Foundation for Angelman Syndrome Therapeutics (FAST), Austin, TX USA; 3 Department of Pediatrics, Duke University School of Medicine, Durham, NC USA

**Keywords:** Angelman syndrome, Reliability, Generalizability theory, Communication, Caregivers

## Abstract

**Aims:**

Caregivers rate improved communication ability as one of the most desired outcomes for successful interventions for individuals with Angelman syndrome (AS). When measuring communication ability in clinical trials, the reliability of such measures is critical for detecting significant changes over time. This study examined the reliability of the Observed-Reported Communication Ability (ORCA) measure completed by caregivers of individuals with AS.

**Methods:**

The ORCA measure was completed by 249 caregivers with 170 caregivers completing the ORCA measure again after 5–12 days. Generalizability theory was used to examine the following sources of measurement error in ORCA scores: concepts, subdomains, assessment points, and the interactions among those facets and the object of measurement: communication ability. Three generalizability studies were conducted to understand the reliability of the ORCA measure for different measurement designs. Decision studies were carried out to demonstrate the optimization of measurement procedures of the ORCA measure.

**Results:**

G and Phi coefficients of the original measurement design exceeded the 0.80 threshold considered sufficiently reliable to make relative and absolute decisions about the communication ability of individuals with AS based on their caregivers’ observed scores. The optimization procedures indicated that increasing the number of communication concepts and/or assessment points leads to more reliable estimates of communication.

**Conclusion:**

The ORCA measure was able to reliably distinguish different levels of communication ability among individuals with AS. Multiple assessment points and or more concepts would provide more precise estimates of an individual’s communication ability but at the cost of survey fatigue.

## Introduction

For caregivers of individuals living with Angelman syndrome (AS), improvement in communication ability is the top priority of a successful therapy or intervention [1]. As a genetic and neurological disorder, individuals with AS tend to have severe developmental delays, profound speech impairment, intellectual disability, and problems with movement and balance, among other symptoms [2]. Taking care of individuals with AS can be challenging because of their impaired communication ability and developmental delay [1]. Communication deficits make it difficult for caregivers to understand their child’s needs for activities of daily living, as most individuals will not achieve verbal speech in their lifetime. Being unable to successfully communicate their needs impacts the quality of life of caregivers and individuals living with AS. The Observer-Reported Communication Ability (ORCA) measure was designed specifically for use in clinical trials of individuals living with AS and is based on caregivers’ observations and ratings [3, 4].

The reliability of any measure used in a clinical trial is critical to detect meaningful and significant changes over time [5]. For instance, to make comparative decisions, researchers must make relative inferences [6], in which participants in the treatment arm are compared with those in the control arm. Absolute inferences are when researchers make generalizations about whether or not the impact of treatment on participants’ outcomes is statistically supported [6]. The accuracy of such decisions is built on the quality of survey measures to yield scores from which reliable generalizations can be made about a participant’s outcome, in this case, their communication ability.

However, achieving adequate score reliability is quite challenging because there are multiple sources of measurement error (i.e. imprecision) associated with the measurement procedure [7]. Reliability is defined as the consistency of the scores across replications of a measurement procedure [8]. Possible sources of measurement error could include (1) the questions on survey instruments, (2) assessment points (i.e., occasions), (3) raters, (4) the interactions among questions, assessment points, and raters, and (5) other hidden facets not accounted for by these sources, such as data collection sites, trial conditions, and modes of data collection. For instance, caregiver-reported measures, such as ORCA and Communication and Symbolic Behavior Scales Developmental Profile Infant-Toddler Checklist (CSBS-DP-ITC), are used in scenarios where caregivers answer all the survey questions within subdomains and repeat the assessment over multiple time points to assess change (e.g., baseline, post-treatment, and long-term follow-up). Each of the five possible sources of measurement error will add a certain amount of imprecision to make relative or absolute inferences. One study applying generalizability theory to CSBS-DP-ITC showed that the questions facet of CSBS-DP-ITC accounted for 47% of the total variance in an AS sample, which brings a substantial amount of imprecision to the scores inference [9].

Thus, a more thorough exploration of reliability, one that goes beyond internal consistency and test re-test reliability, would be useful to establish there is sufficient reliability to use scores for different types of inferences of communication measures within a clinical trial. Multiple guidelines require internal consistency and test-retest reliability coefficients as evidence to support the reliability of clinical outcome assessments (COAs) [10, 11]. The publisher of the CSBS-DP-ITC reported internal consistency coefficient for the subscale scores and total raw scores ranging from 0.87 to 0.93 and test-retest reliability coefficients for raw scores and normative subscale scores ranging from 0.65 to 0.88 [12]. Internal consistency or test-retest reliability represents some aspects of reliability [13] but misses other aspects of reliability that are important for assessing change in psychological measurement within the context of a clinical trial [5]. The internal consistency coefficient focuses on the consistency of scores across all the items and is similar to the average correlation among all item pairs [14]. Test-retest reliability coefficient concerns the agreement between scores that are measured across two time points with the assumption that ability has not changed in the cohort. Neither of these reliability coefficients fully captures the measurement procedure aligned with the intended use of communication measures in clinical trials, which include multiple sources of measurement error.

Therefore, the current study uses generalizability theory to evaluate and investigate the generalizability of scores produced by the ORCA measure. Compared to classical test theory, generalizability theory offers a more comprehensive framework to disentangle multiple sources of measurement error associated with the measurement procedure through the analysis of variance (ANOVA) procedure. The key concepts of generalizability theory are universes of admissible observations and G (Generalizability) studies, universes of generalization, and D (Decision) studies [6, 7]. In a measurement design, universes of admissible observations consist of the facets in measurement procedures and G studies estimate the variance components associated with sources of measurement error. Universes of generalization concern the measurement procedure in future assessment, and D studies evaluate different forms of reliability coefficients and optimize the measurement procedures associated with specified universes of generalization [6, 7, 15].

In the current study, we compare the generalizability and dependability of scores among three measurement designs that are commonly used in clinical research including (1) a reporter-by-concepts design with communication ability as a single dimension, (2) a reporter-by-concepts design with communication ability as multiple subdomains, and (3) a reporter-by-concepts by multiple assessment points design (treating communication ability as unidimensional). The first reporter-by-concepts design is suitable for studies where each caregiver completes the ORCA measure on a single assessment point and produces a single overall score of communication ability. The second reporter-by-concepts within subscale design is appropriate when investigators are interested in the precision of ORCA scores for multiple subdomains of communication (i.e., expressive, receptive, pragmatic communication, and verbal) on a single assessment point. The third design is appropriate for research studies that plan to assess changes in communication ability over time using the ORCA measure. The following research questions (RQs) were proposed:

RQ1: What are the sources of measurement error (i.e., communication concepts, communication subdomains, assessment points) contributing to the imprecision of ORCA measures? Do the contributing sources of error differ among the three measurement designs?

RQ2: How reliable are ORCA measures across the three measurement designs?

RQ3: How can we obtain reliable estimates of ORCA measures when manipulating the number of communication concepts and assessment points in a clinical trial?

## Methods

### Participants

Full details of the validation study are provided elsewhere [3]. The ORCA measure was completed via the web by 249 caregivers of individuals with AS at baseline, and 170 of them completed the ORCA measure again after 5–12 days. There was no expectation that the child’s communication abilities would have changed in this short period; the repeated assessment was performed to evaluate test-retest reliability [3]. The sample of caregivers included 88.2% females, with an average age of 41.6 years [3]. The range of individuals with AS were from 2 to 39 years old, with an average age of 10.5 years. About 67.1% of individuals’ genotype were deletion positive, 20.5% of them had the mutation of the UBE3A gene, 8.4% of them had the UPD, and 4% of them had imprinting center defect. The validation study was approved by the University’s Institutional Review Board.

### Measure

The ORCA measure was designed to be completed independently by a caregiver and assesses different aspects of communication including expressive, receptive, pragmatic, and verbal forms of communication. There are 69 scored survey items organized into 23 communication concepts. The transformation of item responses to communication concept scores was determined through input from caregivers, speech pathologists, and researchers. As a result, 9 concepts (e.g., seek attention, ask questions, and refuse object) were classified as expressive communication, 4 concepts (e.g., greeting, play games, and use names) were classified as pragmatic communication, 8 concepts (e.g., respond to name, respond to questions, and make choices) were classified as receptive communication, and 2 concepts assess the number of words and symbols on an Augmentative and Alternative Communication device their child used. Due to the unique conceptual feature of each communication concept, the range of scores for each concept also differs. There are 3 communication concepts with six levels (scores range from 0 to 5), 5 concepts with five levels (0 to 4), 4 concepts with four levels (0 to 3), 7 concepts with three levels (0 to 2), and 4 concepts with two levels (0 to 1). Concept scores were used as the dependent variables in the generalizability analyses; consistent with the scoring approach of the ORCA measure.

As reported in the previous psychometric study of the ORCA measure [3], the results of confirmatory factor analyses showed that a one-factor model yielded good model fit: comparative fit index (CFI) = 0.96; Tucker-Lewis Index (TLI) = 0.95; Root Mean Squared Error of Approximation (RMSEA) = 0.06, 90% CI: 0.05–0.07. Known-group validity evidence was established based on the fact that individuals with deletion positive genotype had significantly lower ORCA scores than the other genotypes. The strong association between the ORCA measure and CSBS-ITS total scores supported the convergent validity of the ORCA measure. The internal consistency of ORCA scores was α = 0.90 and the test-retest reliability coefficient was ICC = 0.91 with a confidence interval from 0.88 to 0.93.

### Analytic method

To answer the first research question, G studies evaluated the variance of measurement errors and the percentage of variance in total variance for each measurement design. To answer the second research question, we calculated G coefficients, Phi coefficients, relative error variance, and absolute error variance in D studies for each measurement design. G coefficients describe the degree of reliability for making relative decisions or interindividual comparisons, in which individuals are compared with one another in a rank-ordered fashion. Relative error variance is the measurement error associated with G coefficients. Phi coefficients describe the degree of reliability for making absolute inferences or intraindividual comparisons, in which generalizations are made about an individual’s true level of communication ability. Absolute error variance is the measurement error associated with Phi coefficients. The square root of relative or absolute error variance is the standard error of measurement (SEM) for making relative or absolute decisions. To answer the third research question, we demonstrated the optimization procedures through a series of D studies of Model 3, p x I x O, by manipulating the number of communication concepts (I) and the number of assessment points (O). The calculations of variance components were estimated using SAS PROC MIXED with restricted maximum likelihood (REML). REML was selected as the estimation method because it is recommended over ANOVA-based mean squares in situations involving missing data or unbalanced designs [16, 17].

#### Reporter-by-concepts design with communication ability as a single dimension

In a *p x i* model, caregivers’ perceptions, p, the object of measurement, were randomly sampled from the universe of observations (i.e., the population of caregivers) and were administered questions related to the 23 communication concepts (i). The communication concept facet, i, is a random facet because it was treated as the lowest level of measurement in this study and were theoretically selected from an infinite pool of similar concepts [6, 18]. The G coefficient of this model is the analog to the internal consistency coefficient in classical test theory and is written as [6]:

$${\varvec{E}\rho }^{2}=\frac{{\sigma }^{2}\left(\tau \right)}{{\sigma }^{2}\left(\tau \right)+{\sigma }^{2}\left(\delta \right)}=\frac{{\sigma }_{p}^{2}}{{\sigma }_{p}^{2}+\frac{{\sigma }_{pi}^{2}}{{n}_{i}}}$$

The formula for the Phi coefficient of this model is expressed as:

$$\varPhi =\frac{{\sigma }^{2}\left(\tau \right)}{{\sigma }^{2}\left(\tau \right)+{\sigma }^{2}\left({\Delta }\right)}=\frac{{\sigma }_{p}^{2}}{{\sigma }_{p}^{2}+\frac{{\sigma }_{i}^{2}}{{n}_{i}}+\frac{{\sigma }_{pi}^{2}}{{n}_{i}}}$$

#### Person-by-concepts design with communication as multiple subdomains

The second model, *p* x *( i: h)*, is an unbalanced mixed model in which caregivers (*p*) crossed with communication concepts (*i*) nested within four subdomains of ORCA measure (i.e., expressive, receptive, pragmatic, and verbal), with the concept (*i*) as a random facet and communication subdomain (*h*) as a fixed facet. The communication subdomain facet (*h*) was considered as fixed because the four domains encompass all aspects of communication ability we are interested in this study [6, 18]. Due to unequal number of communication concepts (*i*) within each subdomain (*h*), the design is unbalanced. The G coefficient of this model is written as [6]:

$${\varvec{E}\rho }^{2}=\frac{{\sigma }^{2}\left(\tau \right)}{{\sigma }^{2}\left(\tau \right)+{\sigma }^{2}\left(\delta \right)}=\frac{{\sigma }_{p}^{2}+\frac{{\sigma }_{ph}^{2}}{{n}_{h}}}{{\sigma }_{p}^{2}+\frac{{\sigma }_{ph}^{2}}{{n}_{h}}+\frac{{\sigma }_{pi :h}^{2}}{{n}_{i}{n}_{h}}}$$

The formula for the Phi coefficient of this model is expressed as [6]:

$$\varPhi =\frac{{\sigma }^{2}\left(\tau \right)}{{\sigma }^{2}\left(\tau \right)+{\sigma }^{2}\left({\Delta }\right)}=\frac{{\sigma }_{p}^{2}+\frac{{\sigma }_{ph}^{2}}{{n}_{h}}}{{\sigma }_{p}^{2}+\frac{{\sigma }_{ph}^{2}}{{n}_{h}}+\frac{{\sigma }_{pi:h}^{2}}{{n}_{i}{n}_{h}}+\frac{{\sigma }_{i:h}^{2}}{{n}_{i}{n}_{h}}}$$

#### Person-by-concepts by multiple assessment points design (treating communication as unidimensional)

The third model, *p* x *i* x *o*, represented caregivers (*p*) answering questions related to the 23 communication concepts (*i*) on two assessment points (*o*, assessment points at baseline and 2-week follow-up), with the concept (*i*) as a random facet and occasion (*o*) as a random facet as well. The G coefficient of this model is written as [6]:

$${\varvec{E}\rho }^{2}=\frac{{\sigma }^{2}\left(\tau \right)}{{\sigma }^{2}\left(\tau \right)+{\sigma }^{2}\left(\delta \right)}=\frac{{\sigma }_{p}^{2}}{{\sigma }_{p}^{2}+\frac{{\sigma }_{po}^{2}}{{n}_{o}}+\frac{{\sigma }_{pi}^{2}}{{n}_{i}}+\frac{{\sigma }_{pio}^{2}}{{n}_{i}{n}_{o}}}$$

The formula for the Phi coefficient of this model is expressed as [6]:

$$\varPhi =\frac{{\sigma }^{2}\left(\tau \right)}{{\sigma }^{2}\left(\tau \right)+{\sigma }^{2}\left({\Delta }\right)}=\frac{{\sigma }_{p}^{2}}{{\sigma }_{p}^{2}+\frac{{\sigma }_{i}^{2}}{{n}_{i}}+\frac{{\sigma }_{o}^{2}}{{n}_{o}}+\frac{{\sigma }_{po}^{2}}{{n}_{o}}+\frac{{\sigma }_{pi}^{2}}{{n}_{i}}+\frac{{\sigma }_{pio}^{2}}{{n}_{i}{n}_{o}}}$$

Conceptual Explanation of Models Chosen:

Model 1 *(p x i)* and 2 *(p x (i: h))* focused on the measurement procedure that occurs at one-time point and Model 3 *(p x i x o)* related to the longitudinal assessment of communication ability. Model 1 and 3 treats the ORCA score as a unidimensional factor and Model 2 treats the ORCA measure as a multidimensional measure based on the four communication subdomains (expressive, receptive, pragmatic, word/symbol use).

## Results

RQ1: Sources of Measurement Error Contributing to The Imprecision of ORCA Scores.

In model 1, caregivers (p) accounted for over 19% and the concept (i) facet explained about 34% of the total variance (Table 1). The interaction term, caregiver-by-concept was the largest variance component and explained about 47% of the total variance. In model 2, the caregiver-by-subdomain term explained only 2% of the total variance, and subdomain variance was negligible, suggesting that the subdomain facet does not contribute much to the variability in observed scores. Model 3 demonstrated the measurement procedure of assessing communication skills longitudinally or repeatedly. The caregiver explained about 18% and the concept facet accounted for 32% of the total variance. The interaction between caregiver, concept, and occasion explained about 23% of the total variance. The estimates of occasion, caregiver-by-occasion, and concept-by-occasion variance components were minimal, indicating the measurement structure of the ORCA measure is quite stable.

**Table 1:** Variance components and percentage of variance for ORCA scores across three models

Model 1: *p* x i			Model 2: *p* x ( i: h)			Model 3: *p* x i x o		
Facet	Variance	**%**	Facet	Variance	**%**	Facet	Variance	**%**
*p*	0.272	19%	*p*	0.267	18%	*p*	0.252	18%
*i*	0.493	34%	*i: h*	0.493	34%	*i*	0.436	32%
*pi*	0.678	47%	*pi: h*	0.661	46%	*pi*	0.347	25%
			*ph*	0.025	2%	*pio*	0.307	23%
			*h*	0.000	0%	*po*	0.014	1%
						*io*	0.005	0%
						*o*	0.004	0%

*Note N* = 249. *p* = caregiver reporting on communication ability of their child. *i* = communication concepts included on ORCA measure. *o* = assessment points (# of assessment points). *h* = subdomains of communication (e.g., expressive, receptive, pragmatic, and verbal form communication)

RQ2: The Quality of Measurement Design.

For Model 1, G and Phi coefficients were 0.90 and 0.84, which are close to or exceeded the 0.80 threshold considered sufficiently reliable to make decisions about individuals based on their observed scores (Table 2). The G coefficient of model 1 was identical to the internal consistency coefficient in the validation study of ORCA as the G coefficient is analog to the internal consistency coefficient. When including subdomain as a fixed facet in Model 2, the G and Phi coefficients were similar, 0.91 and 0.85, because the average of the caregiver-by-subdomain facet component was negligible. The G and Phi coefficients of model 3 were 0.90 and 0.84, suggesting ORCA scores are quite stable across two assessment points.

**Table 2:** Reliability Coefficients of the Original Measurement Design of ORCA Measure

	Model 1: *p* x i	Model 2: *p* x ( i: h)	Model 3: *p* x i x o
***Eρ*** ^***2***^	**0.902**	**0.905**	**0.898**
*σ* ^*2*^ *(δ)*	0.029	0.029	0.029
***Φ***	**0.842**	**0.845**	**0.836**
*σ* ^*2*^ *(Δ)*	0.050	0.050	0.049

*Note N* = 249. *p* = caregiver reporting on communication ability of their child. *i* = communication concepts included on ORCA measure. *o* = assessment points (# of assessment points). *h* = subdomains of communication (e.g., expressive, receptive, pragmatic, and verbal form communication)

RQ3: Demonstrating the Optimization.

**Fig. 1:**
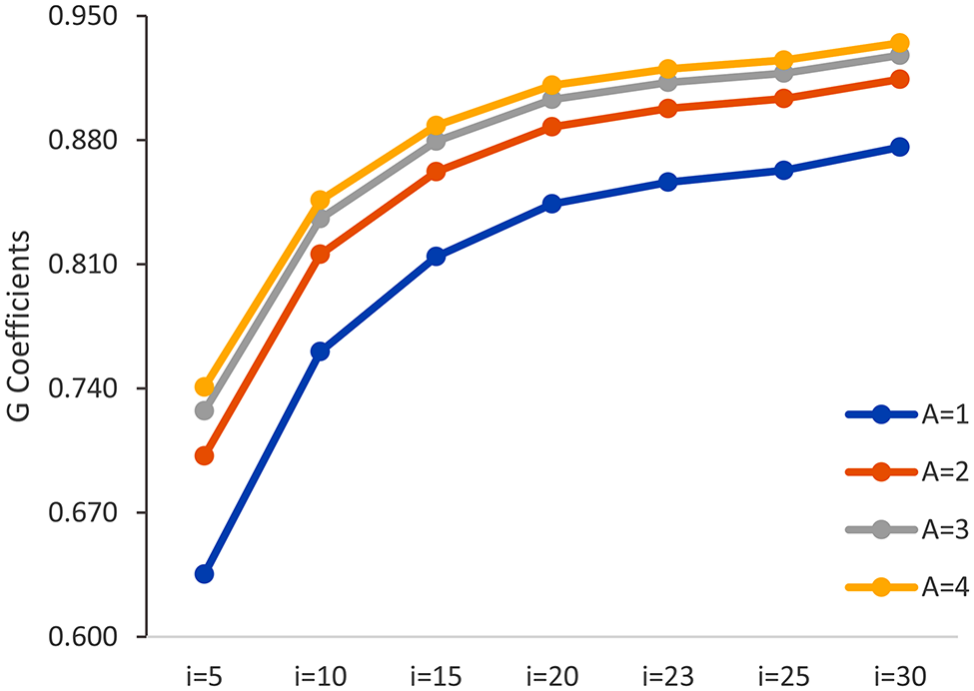
Change of G coefficients when manipulating the number of communication concepts (*I*) and assessment points (*O*)

**Fig. 2:**
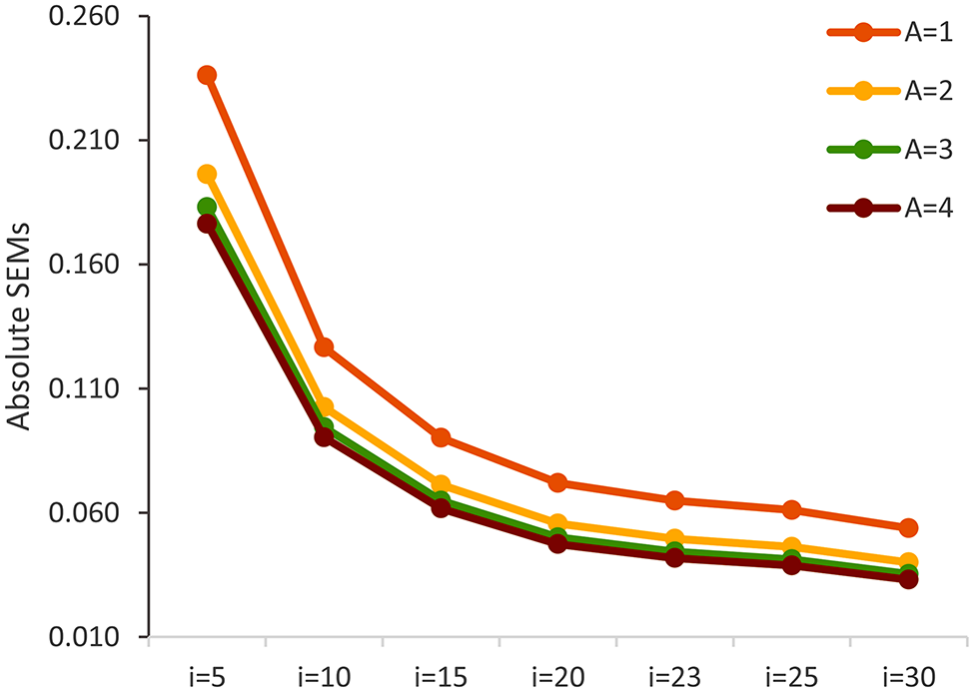
Change of Absolute SEM when manipulating the number of communication concepts (*I*) and assessment points (*O*)

Figures 1 and 2 present the optimization of measurement procedures when manipulating the number of communication concepts and assessment points. Figure 1 illustrates how the G coefficients increase with more communication concepts (from 5 to 25) and the number of assessment points (occasions from 1 to 4) in the ORCA measure (based on the unidimensional model). Figure 2 shows how the absolute SEM reduces with the increasing number of communication concepts and assessment points. Increasing the number of concepts and/or assessment points leads to more reliable estimates of communication ability.

## Discussion

The ORCA measure was designed to be used as an outcome measure of communication ability for non-verbal individuals to estimate treatment benefits in clinical trials for individuals with AS pre/post intervention. As with any outcome measure, it is critical that the ORCA measure has sufficient reliability to identify either changes within a single sample over time (e.g., between baseline and post treatment) or between two different arms (e.g., treatment and control arms) in a randomized trial. The traditional approaches for assessing the reliability of the ORCA measure showed acceptable reliability in terms of internal consistency and test re-test reliability (internal consistency, α = 0.90; test-retest reliability, ICC = 0.91), but a more detailed exploration of the sources of measurement error could help trialists optimize their study design [3]. In addition, a confirmatory factor analysis found sufficient evidence for assessing communication ability as a unidimensional construct (CFI = 0.96; TLI = 0.95; RMSEA = 0.06). This study demonstrated the additional benefits of generalizability theory both for a deeper evaluation of sources of variation as they relate to reliability within a unidimensional and multidimensional framework, and for further enhancements of the use of the ORCA measure in terms of length of the measure and number of assessment points.

With reference to the first research question, the results of the G studies across measurement designs are consistent. The largest variance components were the interaction between communication concepts and object of measurement and the concept facet across designs. Although the concept brings the largest amount of imprecision, it aligns with the intended purpose of the ORCA measure; each concept represents a unique aspect of communication. The desired object of measurement, caregiver perceptions of communication ability of the individual with AS, also explained a fair amount of total variance. The subdomain facets (e.g., expressive, receptive, pragmatic, and verbal communication) and the interaction between subdomains and other facets were negligible, which provides additional support for the unidimensional structure of the ORCA measure within the AS population. For the third design, the assessment point facet (occasions) and the interaction between it with caregiver were minimal, which supports that the ORCA measure has a stable measurement structure over repeated assessments of communication ability.

The results of the D studies supported that ORCA scores are consistent across models. It indicates that the ORCA measure yields scores that can support reliable relative and absolute inferences regarding caregivers’ perceptions of the communication ability of individuals with AS at a considerable reliable criterion (> 0.80) [16]. The D study results also showed how score precision improves when including more communication concepts and number of assessment points. This implies that by incorporating a comprehensive range of communication concepts and conducting multiple assessments, the ORCA measure becomes even more precise in gauging caregivers’ perceptions, enhancing the overall reliability and validity of the scores obtained. However, the gain in precision needs to be considered in contrast to survey fatigue caregivers will experience filling out the survey with more concepts or multiple times.

The results of this study must be interpreted with the following limitations. First, the intended use of the ORCA measure is to evaluate the communication ability of individuals with AS. If using the ORCA measure with individuals with other neurodevelopmental disorders, the reliability of the ORCA scores may vary, and adjustments of survey questions might need to be made for certain populations. In addition, the stability of the ORCA scores in longitudinal assessment was based on two assessment points in a psychometric study designed to establish evidence for test-retest reliability. Future research is needed that investigates the generalizability of ORCA scores in studies with group comparisons (e.g., control vs. treatment group) and with more assessment points after an intervention. Finally, while our sample was a comprehensive representation of different age groups and AS genotypes, the majority of caregivers included in this study were Caucasian and had some level of college or graduate education. In future research, it is crucial to establish the reliability and validity evidence of ORCA of all families, particularly those who are disproportionately affected by institutionalized and structural racism.

## Conclusions

The current study used generalizability theory to evaluate the reliability of the ORCA measure across multiple models incorporating difference sources of error variance. Subsequent procedures can then be designed to reduce measurement errors that contribute the most to imprecision and optimize the generalizability of measurement. The results of our study are consistent with other studies applying generalizability theory to communication measures in individuals with AS [9], which found that concept variance accounted for the majority of total variance, and the object of measurement explained a fair amount of total variance. However, our study improves on previous findings by distinguishing the influence of subdomains and assessment points. We found that although a single assessment point offers sufficient reliability, adding more concepts or multiple administrations would improve the precision of estimates of communication ability.

## Data Availability

The datasets generated and/or analyzed during the current study are not publicly available due to the sensitive nature of the research. The participants of this study did not give written consent for their data to be shared publicly.
